# Acoustic Source Drone Detection System Using Tetrahedral Microphone Array and Deep Neural Networks

**DOI:** 10.3390/s26061778

**Published:** 2026-03-11

**Authors:** Marian Traian Ghenescu, Veta Ghenescu, Serban Vasile Carata

**Affiliations:** 1 Institute of Space Science-Subsidiary of INFLPR, 409 Atomistilor Street, 077125 Magurele, Romania; marian.ghenescu@spacescience.ro; 2 Onest Solutions, 077191 Voluntari, Romania

**Keywords:** acoustic localization, microphone arrays, drone detection, source localization, robust localization, deep learning, sensor fusion

## Abstract

The rapid integration of Unmanned Aerial Vehicles (UAVs) into civilian airspace has introduced complex security challenges, particularly regarding the protection of critical infrastructure and personal privacy. While conventional detection mechanisms such as radar and optical sensors are widely deployed, they are frequently limited by line-of-sight obstructions and the small radar cross-section of modern commercial drones. Acoustic analysis presents a viable passive alternative; however, accurate three-dimensional localization remains a computationally demanding task, further complicated by the use of directional sensors with non-uniform sensitivity patterns. In this paper, a deep learning framework is proposed to address these ambiguities. The method involves the fusion of raw acoustic data with explicit sensor geometry metadata within a neural network architecture. To enhance localization precision, a composite loss function is introduced, which independently optimizes planar and altitude coordinates while penalizing outlier predictions. Experimental validation was conducted using a custom dataset of real-world drone flights, utilizing a distributed array of directional microphones. The results demonstrate that the proposed system effectively mitigates the spatial irregularities of ad hoc sensor deployment, achieving robust localization performance in complex acoustic environments.

## 1. Introduction

In the contemporary security landscape, the accessibility of Unmanned Aerial Vehicles (UAVs) has fundamentally altered the requirements for airspace monitoring. The potential for misuse—ranging from the unauthorized surveillance of private property to the disruption of operations at airports and industrial facilities—necessitates the deployment of robust Detection, Tracking, and Identification (DTI) systems. The “drone threat” is characterized by asymmetry. Low-cost, commercially available devices can often bypass expensive, traditional security perimeters designed for larger aircraft. Consequently, both governmental and industrial entities have prioritized the development of effective countermeasures.

Current detection strategies heavily rely on active radar, cameras, and Radio Frequency (RF) scanners. However, these methods have inherent physical limitations. Radar systems, particularly in urban environments, often struggle to distinguish small, plastic drones from biological targets such as birds, or from ground clutter (the “urban canyon” effect). Optical systems require a clear line of sight, and their performance is significantly reduced in adverse weather conditions, including fog and heavy rain, as well as in low-light environments. RF scanners, while effective against commercial drones utilizing standard communication protocols, are rendered ineffective by autonomous drones operating on pre-programmed waypoints without active radio links.

Unlike active acoustic sensing systems (e.g., sonar or echolocation-based methods like VoiceMap or CATS) which emit sound waves and analyze reflections—a process that requires significant power and can reveal the detector’s location—our proposed system is entirely passive. It relies solely on the emitted acoustic signature of the target drone. This passive nature is critical for security scenarios where the detection system must remain covert. Furthermore, while systems like WSTrack focus on tracking active acoustic tags, our approach targets the inherent mechanical noise of the drone rotors, requiring no modification to the target vehicle.

In this context, acoustic sensing emerges as a critical, complementary technology. It functions passively, requires no line of sight, and targets the unique, unavoidable noise signature generated by the UAV’s propulsion system. However, the transition from simple acoustic detection (identifying the presence of a drone) to precise 3D localization (determining its coordinates) presents substantial engineering challenges. Acoustic detection and localization are closely related but conceptually distinct tasks within acoustic signal processing [1]. Acoustic detection refers to the process of identifying sound signals present in the environment, whereas acoustic localization concerns determining the spatial position of the source generating that sound [2]. These techniques are widely used across numerous domains, both military and civilian—for example, in robotics [3,4], search-and-rescue operations [5,6], and underwater or maritime detection [7,8]. These represent only a subset of the many application areas in which acoustic detection and localization play central roles, often operating in parallel with video-based detection and localization methods [9,10,11]. Integrating both acoustic and visual sensing modalities typically leads to significantly improved localization accuracy.

Specifically, to achieve the Signal-to-Noise Ratio (SNR) required for long-range detection, modern systems frequently employ directional microphones. Unlike omnidirectional sensors, which exhibit uniform sensitivity, directional microphones introduce a complex dependency between the source’s position and the recorded signal intensity. The amplitude of the signal is determined not only by the distance (following the inverse square law) but also by the angle of arrival relative to the microphone’s sensitivity lobes. This introduces spatial ambiguities that classical geometric algorithms—such as Time Difference of Arrival (TDOA)—often fail to resolve accurately [12,13].

This paper addresses these limitations through the proposal of a multimodal deep learning framework. The hypothesis is that by explicitly encoding the physical state of the sensor array (position, heading, and tilt) and fusing this metadata with spectral audio features, a neural network can learn to compensate for the directional characteristics of the sensors. Furthermore, the specific problem of “overshoot” in 3D localization—where altitude estimation errors degrade the overall tracking quality—is addressed by implementing a composite loss function that applies differential weighting to planar and vertical coordinates.

The remainder of this paper is structured as follows: Section 2 reviews the state of the art in acoustic localization. Section 3 details the experimental hardware and dataset generation. Section 4 elaborates on the neural network architecture and fusion strategy. Section 5 presents the experimental validation and trajectory analysis, followed by conclusions in Section 6.

## 2. Related Work

The domain of acoustic source localization has evolved from purely physics-based signal processing to data-driven learning approaches. This section categorizes existing literature and identifies the specific technical gaps addressed by the present research.

### 2.1. Classical Signal Processing Approaches

For decades, the standard for Sound Source Localization (SSL) has been grounded in geometric algebra. Techniques such as Time-Difference-of-Arrival (TDOA) and Generalized Cross-Correlation (GCC) rely on estimating the propagation delay of a wavefront across a spatially distributed array. By solving a system of hyperbolic equations, the source position can theoretically be triangulated. Alternatively, beamforming techniques, including Steered-Response Power (SRP) and Minimum Variance Distortionless Response (MVDR), spatially filter the environment to identify energy peaks corresponding to the source.

While theoretically sound, these methods rely on simplifying assumptions: that the environment is anechoic, that the noise floor is stationary, and that the microphones act as ideal point receivers. In real-world operational scenarios—characterized by wind noise, multipath reflections from buildings, and the broadband spectral nature of drone rotors—these assumptions frequently break down [14,15,16,17]. Consequently, the accuracy of classical solvers degrades significantly, often resulting in unstable localization fixes or “ghost” targets.

### 2.2. Challenges with Directional Sensors

The integration of directional microphones adds another layer of complexity to the localization problem. Standard TDOA formulations assume that the signal amplitude and phase are consistent across the array, modified only by the propagation path. However, directional sensors (e.g., cardioid or shotgun microphones) impose a spatial filtering function on the input. A source located off-axis will be attenuated, potentially confusing energy-based localization algorithms. As noted in [18,19], non-uniform array geometries and directional patterns can create “blind spots” and increase the likelihood of spatial aliasing. This is particularly problematic for 3D localization, where the *z*-axis (altitude) estimation is highly sensitive to amplitude variations. If a drone flies directly above a directional microphone that is pointed towards the horizon, the signal drop might be misinterpreted as the drone flying away, rather than flying up.

### 2.3. Deep Learning and Sensor Fusion

The advent of deep learning has facilitated a shift toward end-to-end localization models. Convolutional Neural Networks (CNNs) have proven highly effective at extracting features from time-frequency representations (spectrograms), implicitly learning to suppress reverberation and background noise. Recent studies in the UAV domain have utilized Deep Neural Networks (DNNs) for Direction-Of-Arrival (DOA) regression [20].

However, a critical gap remains in the literature: most existing deep learning models treat the microphone array as a static, “black box” input. They do not explicitly account for the physical configuration of the sensors as a dynamic variable. To capture the acoustic data, we utilized a tetrahedral array of *Brüel & Kjær* Type 4958 microphones. These sensors were selected specifically for their excellent phase-matching characteristics (<$2^{\circ }$ up to 5 kHz) and uniform magnitude response, which are critical for accurate Time Difference of Arrival (TDOA) estimation in array processing. The compact ½-inch form factor minimizes diffraction effects around the microphone body in the frequency range of interest (200 Hz–4 kHz). A critical aspect of our data collection methodology was the variation of sensor geometry. To ensure the neural network learned to generalize spatial features rather than overfitting to a specific array shape, the microphones were not fixed in a static rigid body. Instead, the microphones were physically moved and reconfigured between each distinct data gathering run. The relative distances ($d_{ij}$) and the orientation of the tetrahedron were varied, and these specific geometry coordinates were fed as metadata into the network alongside the audio data. This dynamic configuration prevents the model from memorizing a single spatial transfer function.

Furthermore, few studies address the full 3D localization problem (x, y, z) in outdoor environments, often restricting the scope to 2D angular estimation (azimuth and elevation) or ground-plane tracking [21]. Our work seeks to bridge this gap by treating the sensor geometry not as a fixed hyperparameter, but as a dynamic input vector fused with the audio stream, allowing for ad hoc array deployment.

## 3. Methodology and Data Acquisition

A prerequisite for training robust localization models is the availability of a dataset that captures the stochastic nature of real-world acoustic environments. Since no publicly available dataset met the specific requirements for directional microphones and variable array geometries, a custom experimental setup was constructed.

### 3.1. Hardware Architecture

**Figure 1:**
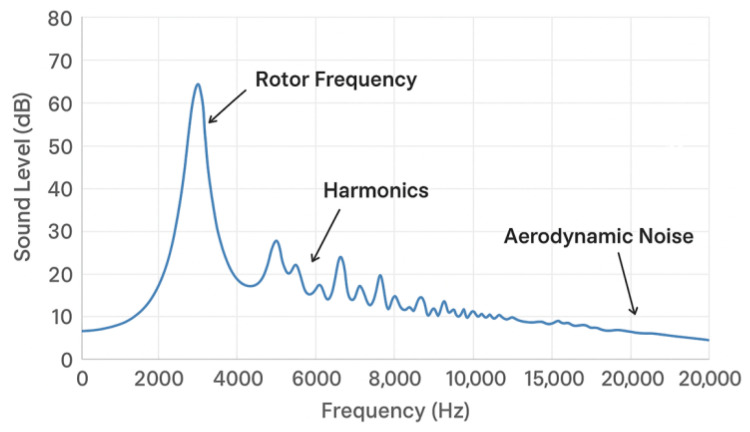
Representative acoustic spectrum of a small quadcopter (DJI Mavic Pro–class).

The choice of sensors for the acoustic signal for drone detection must take into account the specific noise produced by drones-considering here standard commercial drones. Acoustic frequencies of interest based on typical frequencies for drone noise-the noise of a rotor drone (quad-, hexa- or octocopter) is dominated by:

- The main frequency (Blade-Pass Frequency (BPF)). The BPF is the frequency at which the blades pass through the same point. Frequency spectrum: 100–300 Hz, depending on propeller diameter, motor speed (RPM), number of blades.
- Harmonics of the main frequency (noise perceived as a “buzz” or “whir”). Harmonics typically extend up to 1 kHz to 5 kHz (most noticeable to the human ear), sometimes up to 10–15 kHz, depending on propeller design and turbulence.
- High-frequency component (aerodynamic) originating from blade tips and vortices. The frequency spectrum is wider: 5–20 kHz. The signal intensity varies from model to model, especially depending on the size of the drone and the flight regime, but the range of acoustic frequencies of interest is from a few tens of Hz to a few tens of kHz. The acoustic signature of such drones—see Figure 1 is characterized by a harmonic stack structure, where the fundamental frequency $f_{0}$ corresponds to the rotor rotation speed (typically 150–200 Hz for this class of drone) and its harmonics $nf_{0}$ extend up to 5–6 kHz. This spectral content dictates our bandwidth requirements.

**Figure 2:**
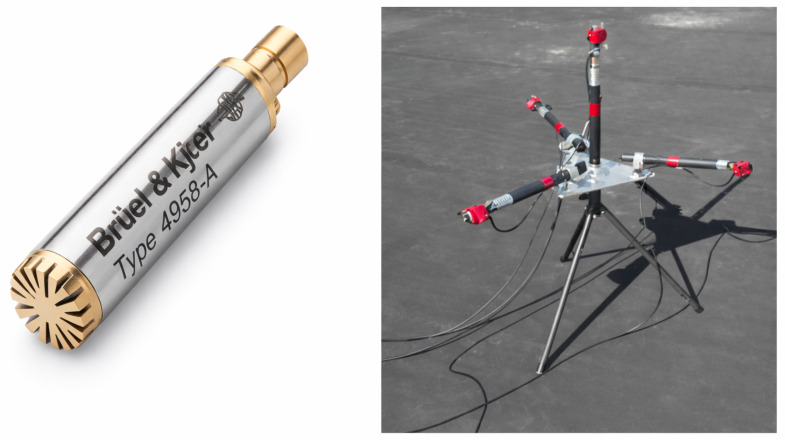
(**left**) *Brüel & Kjær* Type 4958 microphone; (**right**) Image of microphones setup configuration.

Based on this information, we analyzed the acoustic sensors in order to identify the best option for the purpose of our paper. The data acquisition system (see Figure 2) was designed around four high-fidelity directional microphones, specifically the *Brüel & Kjær* Type 4958 [22].

**Figure 3:**
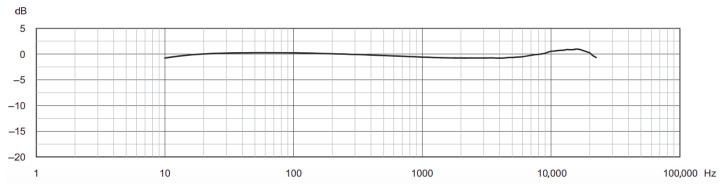
Representative acoustic spectrum of *Brüel & Kjær* Type 4958 microphone.

These are $\frac{1}{4}$-inch prepolarized microphones optimized for array applications. They were selected for their exceptional phase matching and amplitude stability (see Figure 3) across varying temperature and humidity conditions—critical factors for maintaining synchronization in outdoor deployments where environmental factors can induce drift.

The microphones were connected to a National Instruments NI-9234 dynamic signal acquisition module, housed in an NI-USB-9162 chassis. This setup ensured 24-bit resolution with simultaneous sampling, eliminating inter-channel jitter that could otherwise corrupt the TDOA features essential for localization. The synchronization of the audio streams is paramount; even millisecond delays can result in localization errors of several meters. The acoustic source used in this study was a commercial quadcopter (DJI Mavic Pro class). The drone is powered by four electrically driven propellers and produces a characteristic acoustic signature dominated by tonal components associated with the blade-passing frequency and its harmonics, superimposed on a broadband noise floor arising from aerodynamic turbulence and motor operation.

Audio signals were acquired using the NI-9234 at its native sampling rate of 51.2 kHz to ensure capture of the maximum available bandwidth. The raw recordings were subsequently resampled in software to 48 kHz, a standard sampling rate in professional audio and video applications, providing precise control over the sample rate used within the deep learning framework. This resampling preserves the full harmonic content of the drone noise while avoiding aliasing.

Time–frequency analysis was performed using a Short-Time Fourier Transform (STFT) with a Hann window. The window length and FFT size were both set to 2048 samples at a sampling rate of 48 kHz, resulting in a frequency resolution of approximately 23.4 Hz. A hop length of 160 samples was used, corresponding to significant overlap between adjacent frames, ensuring smooth temporal coverage of the signal. The Hann window was chosen to suppress spectral leakage, which is critical for distinguishing tonal drone harmonics from broadband background noise.

During operation, the dominant spectral energy of the drone noise is concentrated in the low-to-mid frequency range, with additional harmonic content extending to higher frequencies depending on flight mode, thrust level, and relative distance to the microphone array. These acoustic characteristics are consistent with those reported in the literature [14] for similar multirotor platforms and informed the selection of the microphones, sampling rate, and time–frequency representation used in this work.

### 3.2. Variable Array Geometry

To prevent the neural network from overfitting to a specific spatial configuration, the microphone array geometry was altered for each flight session. A non-uniform, distributed layout was utilized rather than a compact, symmetrical array.

For every recording, the physical parameters of each microphone were measured and logged:

- **Position:** Relative (x,y) coordinates on the ground plane, established via laser rangefinder.
- **Height:** Sensor elevation (*z*), varying between 90 cm and 170 cm to provide vertical aperture.
- **Heading:** The azimuthal direction the microphone was facing ($0^{\circ }$ to $360^{\circ }$).
- **Orientation:** Whether the microphone was mounted horizontally or vertically.

This variability is central to the proposed methodology. By forcing the model to solve the localization problem across different array shapes, it is ensured that the learned features represent generalized physical principles rather than memorized spatial lookup tables.

### 3.3. Data Collection Protocol

The dataset comprises approximately 40 min of flight time, divided into four distinct sessions. The acoustic source was a commercial quadcopter performing a diverse set of maneuvers designed to probe the limits of the localization system:

1. **Vertical Profiles:** Hovering at incremental altitudes (1 m to 35 m) to generate specific data for altitude regression training.
2. **Planar Transects:** Flying linear paths across the array baseline to capture Doppler shifts and lateral amplitude variations.
3. **Dynamic Trajectories:** Complex, non-linear flight paths (figure-eights, rapid ascents) to simulate evasive maneuvers typical of non-cooperative drones.

The recording environment was chosen to simulate an “operational” edge case. The site included a reflective building facade on one side and a dense forest line on the other. This setup naturally introduced acoustic shadowing, multipath reflections, and wind noise, providing a rigorous test for the system’s robustness against environmental interference.

## 4. System Architecture and Deep Learning Framework

The core of the proposed solution is a multimodal Deep Neural Network (DNN) that performs regression on the 3D coordinates of the target. The architecture is designed to fuse two disparate data modalities: the high-dimensional, temporal audio signal and the low-dimensional, static metadata vector.

### 4.1. Acoustic Feature Extraction

The raw audio streams are first segmented into non-overlapping windows of one second. Given the sampling rate, each window captures sufficient temporal context to characterize the rotor harmonics. These raw waveforms are transformed into the Time-Frequency domain using the Short-Time Fourier Transform (STFT).

We specifically utilize Log-Mel Spectrograms as the input representation. The configuration includes:

- **FFT Size:** 2048 points, providing high frequency resolution to distinguish rotor RPM variations.
- **Hop Length:** 160 samples, ensuring fine temporal granularity.
- **Mel Bins:** 128 bands, covering the frequency range from 100 Hz to 8 kHz.

The resulting tensor (Time × Frequency × Channels) serves as the input to a Convolutional Neural Network (CNN) backbone. This subnet consists of multiple convolutional layers with Batch Normalization and ReLU activation, designed to extract spatial features such as Inter-channel Time Differences (ICTD) and Inter-channel Level Differences (ICLD) directly from the spectral data. The network effectively learns to treat the spectrogram as an image, identifying the distinct “fingerprint” of the drone’s noise against the background.

### 4.2. Metadata Embedding and Fusion

Simultaneously, the sensor geometry is processed via a parallel branch. The 12-dimensional metadata vector (comprising the position and orientation of the four microphones) is passed through a Multi-Layer Perceptron (MLP). This network projects the physical parameters into a higher-dimensional “semantic” embedding space (128 units).

The fusion strategy employs feature concatenation. The flattened feature vector from the audio CNN is merged with the geometry embedding. This combined representation is then processed by a series of fully connected layers. This architecture effectively allows the network to “modulate” its interpretation of the audio features based on the known configuration of the sensors. For instance, if the metadata indicates a specific microphone is facing away from the source, the network can learn to reduce the weighting of amplitude cues from that channel while relying more on phase information.

The Ground Truth (GT) for the drone’s position was captured using the internal drone logging software providing centimeter-level accuracy for training and evaluation. The dataset was partitioned using a strict run-based split to prevent data leakage. We recorded 4 separate flight paths. These were split as follows: 70% of the distinct flight runs were used for Training, 15% for Validation, and 15% for Testing. This ensures that the test results reflect the system’s ability to generalize to unseen trajectories.

### 4.3. Composite Loss Function

A critical contribution of this work is the design of the loss function. In 3D localization, errors in different dimensions have different operational consequences. A 5 m error in horizontal position (x,y) might be acceptable for general monitoring, but a 5 m error in altitude (*z*) when the drone is flying low could lead to a false negative (predicting the drone is on the ground when it is actually airborne). Furthermore, regression models often suffer from “overshoot,” predicting targets at implausible distances when the signal is weak.

To address this, a composite loss function L is proposed, defined as:

(1) $$L=\alpha \cdot L_{\mathrm{planar}}+\beta \cdot L_{\mathrm{alt}}+\gamma \cdot L_{\mathrm{overall}}$$

where:

- $L_{\mathrm{planar}}$ is the Mean Squared Error (MSE, L2 norm) of the horizontal coordinates. The L2 norm penalizes large deviations, encouraging the model to converge quickly on the general location.
- $L_{\mathrm{alt}}$ utilizes the Mean Absolute Error (MAE, L1 norm) for the altitude. The L1 norm is more robust to outliers, preventing the model from reacting violently to transient acoustic anomalies that might suggest extreme altitudes.
- $L_{\mathrm{overall}}$ represents the Euclidean distance in 3D space, ensuring global consistency.

The weighting coefficients (α,β,γ) allow the system’s sensitivity to be tuned, prioritizing planar accuracy while constraining vertical variance.

## 5. Experimental Validation and Discussion

The performance of the system was evaluated using a strict frame-by-frame protocol. No temporal smoothing (e.g., Kalman filtering) or trajectory constraints were applied during inference. Each prediction is the result of a single, independent audio window. This methodology ensures that the reported metrics reflect the raw discriminative power of the neural network, establishing a conservative baseline for system performance.

### 5.1. Localization Accuracy vs. Range

The quantitative results, summarized in Table 1, reveal a distinct relationship between source distance and localization error.

In the near-field region (0–3 m), the error rate is significantly higher (approx. 11–14%). This phenomenon is attributable to the geometric layout of the array. When the source is located within the convex hull of the microphones, small angular deviations in the DOA estimation translate into large relative position shifts. Furthermore, “near-field” acoustic effects (spherical wavefront curvature) are most pronounced here, potentially introducing non-linearities that the model finds challenging.

**Figure 4:**
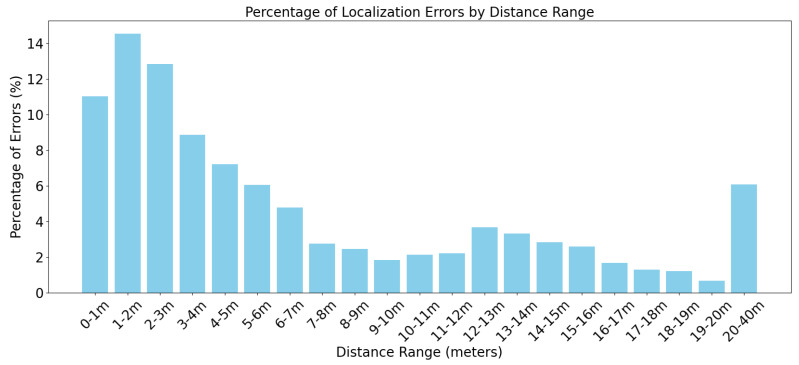
Localization error plotted against distance. The curve illustrates the transition from near-field geometric sensitivity to stable mid-field tracking, followed by a gradual degradation in the far-field due to SNR attenuation.

However, as the target moves into the mid-field (3–10 m), the system stabilizes rapidly. The error percentage drops below 2% in the 9–10 m range, as shown in Figure 4.

At extended ranges (20–40 m), the error rate sees a modest increase to roughly 6%. Considering the environmental factors—wind attenuation, atmospheric absorption, and ground reflections—this retention of accuracy is significant. It demonstrates that the directional microphones, when correctly modeled by the network, successfully preserve the Signal-to-Noise Ratio required for far-field detection.

### 5.2. Trajectory Reconstruction Analysis

**Figure 5:**
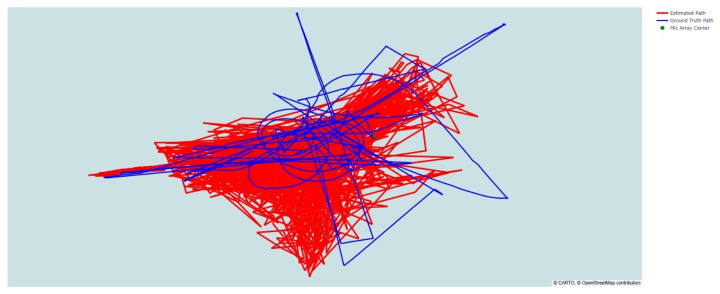
3D Trajectory Tracking Performance. The blue line shows the GT drone trajectory, and the red points represent the raw acoustic estimates. The predictions consistently follow the GT trends, with a small Root Mean Square Error (RMSE), effectively mitigating the altitude overshoot observed in baseline methods.

Beyond aggregate statistics, the system’s utility depends on its ability to reconstruct coherent flight paths. Figure 5 compares the ground-truth (GT) trajectory obtained from GPS/telemetry with the acoustic predictions.

The predicted positions (red) form a dense point cloud centered around the ground-truth path (blue). Importantly, the error distribution appears largely unbiased, with the cloud remaining concentric with the trajectory rather than exhibiting a systematic offset. If the model had failed to account for a microphone’s orientation, we would expect to see “drift” or constant offsets in specific sectors of the airspace.

As observed in Figure 5, the raw network predictions (Red) form a point cloud distributed around the true trajectory (Blue). While there is inherent stochastic variance in instantaneous acoustic estimation (due to wind gusts and momentary SNR drops), the estimates consistently track the drone’s path. In a deployed security operations context, this variance is mitigated by the system’s high temporal resolution.

The feature extraction and neural network inference require approximately 26.63 ms per audio frame on a standard GPU (NVIDIA GeForce RTX 4070M), which is significantly faster than the audio acquisition rate. This low latency allows for the application of a Kalman Filter or Moving Average post-processing stage in real-time, effectively smoothing the point cloud into a continuous trajectory without introducing significant lag.

## 6. Conclusions and Future Work

The research presented in this paper validates the efficacy of a deep learning-based approach to acoustic drone localization using directional microphone arrays. By shifting from static geometric models to a dynamic, data-driven framework, it has been demonstrated that it is possible to achieve robust 3D localization even with irregular sensor configurations and directional ambiguities.

Key conclusions include:

- **Metadata Fusion:** The explicit inclusion of sensor geometry as an input vector is not merely beneficial but essential for handling directional arrays. It allows the network to decouple signal intensity from source distance.
- **Operational Robustness:** The system maintains high accuracy (error rates <3%) in the critical 7–15 m range, making it a viable component for perimeter defense systems.
- **Composite Loss Function:** The separation of planar and altitude error terms during training effectively mitigates the “overshoot” problem common in 3D regression tasks.

Future research will focus on two axes. First, the integration of temporal context through Recurrent Neural Networks (RNNs) or Transformer architectures to inherently smooth the trajectory predictions. Second, the fusion of this acoustic subsystem with low-cost radar or optical sensors to create a fully redundant, multi-spectral Counter-UAS (C-UAS) solution capable of operating in all weather conditions. The dataset generated for this study has been indexed and will be made available to the research community to foster further development in this critical security domain.

## Figures and Tables

**Table 1:** Analysis of localization error distribution as a function of source distance. The system exhibits optimal stability in the 3–15 m operational range.

Distance Range (m)	Error Count	Error Percentage (%)
0–1	529	11.02
1–2	697	14.52
2–3	616	12.83
3–4	425	8.85
4–5	346	7.21
5–6	290	6.04
6–7	230	4.79
7–8	132	2.75
8–9	118	2.46
9–10	88	1.83
10–11	103	2.15
11–12	106	2.21
12–13	176	3.67
13–14	160	3.33
14–15	136	2.83
15–16	124	2.58
16–17	80	1.67
17–18	62	1.29
18–19	58	1.21
19–20	32	0.67
20–40	292	6.08

## Data Availability

The data presented in this study are available on request from the corresponding author.
